# Prevalence of overweight, obesity and central adiposity among grade seven adolescents in urban Tshwane, South Africa

**DOI:** 10.4102/hsag.v31i0.3328

**Published:** 2026-05-26

**Authors:** Nonie M. Mokose, Jan W. Swanepoel, Wilna Oldewage-Theron

**Affiliations:** 1 Department of Sustainable Food Systems and Development, Faculty of Natural and Agricultural Sciences, University of the Free State, Bloemfontein, South Africa

**Keywords:** adolescent obesity, overweight, central adiposity, socioeconomic status, Urban health, South Africa

## Abstract

**Background:**

The global increase in overweight and obesity among adolescents is a significant public health priority, especially in low- and middle-income countries. In South Africa, urbanisation and lifestyle changes have exacerbated this challenge, but there is a lack of disaggregated data available to inform effective local responses.

**Aim:**

To assess and describe the prevalence of overweight, obesity and central adiposity in Grade seven adolescents in urban Tshwane, South Africa, and explore its association with socioeconomic characteristics.

**Setting:**

The study was conducted in five diverse fee-paying public primary schools in urban Tshwane, Gauteng province.

**Methods:**

A quantitative, descriptive cross-sectional design recruited 164 adolescents aged 11–14 years. Anthropometric measures were performed according to World Health Organization (WHO) standards. These included a survey of height and weight to derive body mass index (BMI-for-age) z-scores, as well as waist circumference to height ratio (WHtR). Socioeconomic status was assessed using the validated Family Affluence Scale. Data analysis included descriptive statistics and chi-square tests to determine the association between categorical variables.

**Results:**

The combined prevalence of overweight and obesity was 23.7%, with a greater burden among girls (36.4% of 12 year olds) compared to boys (16.7%). The risk of central adiposity (WHtR ≥ 0.5) was found in 25% of participants, with 7.3% at very high risk. Household income was significantly associated with obesity (*p* = 0.043), while parental education was not statistically significant.

**Conclusion:**

The high prevalence of excess adiposity, particularly among female adolescents, indicates an emerging cardiometabolic risk profile among the group.

**Contribution:**

This study addresses a critical gap in South African public health literature by providing locally specific, disaggregated data on the nutritional status of urban adolescents in Tshwane, a cohort that is underrepresented in contrast to conventional rural or national surveys.

## Introduction

The worldwide occurrence of overweight and obesity among adolescents keeps increasing at a fast pace as a significant public health concern, particularly in low- and middle-income countries (LMICs) such as South Africa. The World Health Organization (WHO 2021) reported that the global prevalence of obesity among children and adolescents has increased almost threefold since 1975, with an excess of 340 million aged 5–19 years being classified as overweight or obese. The development creates major issues for LMICs because their fast urbanisation and economic expansion and dietary pattern changes have triggered a nutrition transition that combines rising energy-dense food consumption with declining physical activity levels (Malik & Hu 2022; Popkin 2001). As one of the most urbanised countries on the sub-continent, South Africa is no exception to the global increase in obesity, with adolescent obesity rates reaching problematic levels (Otitoola, Oldewage-Theron & Egal 2020; Wrottesley et al. 2019). Adolescence is a critical period for establishing health behaviours that persist throughout the lifespan. Consequently, the onset of excess weight during this stage does more than threaten physical health; it can also result in long-term challenges for mental health, self-esteem and social integration (Alberga et al. 2012; Patton et al. 2016). Adolescent obesity is a major risk factor for adult non-communicable diseases (NCDs) including type-2 diabetes (T2D), cardiovascular diseases and specific cancers (De Amicis et al. 2022; Okop, Agabi & Joseph 2025). The double burden of malnutrition (DBM) creates an extra challenge to the health status of South Africans because under- and overnutrition coexist in the same communities and households (Onyango et al. 2019; Shrimpton & Rokx 2012). Rapid urbanisation and the associated transition in lifestyle have intensified the overweight and obesity epidemic among adolescents in South Africa. The obesity epidemic affects urban centres like Tshwane, which serves as South Africa’s administrative capital. Foods that contribute to the obesity problem, such as ultra-processed foods and fast foods, are easily accessible compounded with people spending increasing sedentary time while being less active (Phetla & Skaal 2023; Pineda et al. 2024). This is exacerbated by socioeconomic inequalities, as adolescents from households with limited income levels struggle with food insecurity and limited access to healthy food options, but paradoxically experience greater rates of obesity (Naicker, Mathee & Teare 2015; Otitoola et al. 2020). The public health community acknowledges adolescent obesity as a priority, yet there is insufficient data about overweight and obesity rates among Tshwane’s urban youth population. Although national datasets, such as the South African National Health and Nutrition Examination Survey (Shisana et al. 2013), provide valuable insights on the overall obesity burden, results from national surveys often lack granularity to enable the design of targeted interventions at the local level. The majority of South African research about adolescent obesity studies rural and peri-urban areas, which leaves a knowledge gap regarding the unique challenges urban adolescents face (Ngwenya & Ramukumba 2017; Wrottesley et al. 2019). The current study will help answer the question by establishing the prevalence of overweight and obesity among Grade 7 adolescents in urban Tshwane using BMI as the main indicator. Body mass index is a widely accepted and practical measure for determining weight status in adolescents, as it caters to age- and gender-related growth patterns (Kuzik et al. 2022; WHO 2007). The study will improve current knowledge about adolescent obesity in South Africa by delivering data about overweight and obesity rates in this population, which may help create specific public health interventions. The study aims to determine and describe the prevalence of overweight, obesity and central adiposity among Grade seven adolescents in urban Tshwane, South Africa, and to examine the relationships between the patterns of adiposity and a range of socioeconomic characteristics.

## Research methods and design

### Study setting

The study was conducted in urban Tshwane, South Africa. According to the 2022 Statistics South Africa census, the City of Tshwane has a total population of 4 039 960 (Statistics South Africa 2024). Among this, the early adolescent cohort (aged 11–14 years), which directly corresponds to the study’s target population, represents a significant demographic, providing the context for the school-based sampling of Grade 7 learners). The research focused on English-medium, inner-city public primary schools (Quintiles 4 and 5). While these fee-paying schools do not represent the full socioeconomic spectrum of South Africa, they offer insight into the urban middle class. This cohort is uniquely positioned within the nutrition transition, where purchasing power often coincides with access to ultra-processed foods. The fast urbanisation of Tshwane creates an obesogenic environment through its abundant unhealthy food options, scarce physical activity spaces and social economic inequalities (Phetla, Skaal & Chelule 2024). The situation demonstrates the challenges that low- and middle-income urban areas encounter when addressing adolescent obesity.

### Design

The study was carried out by using a quantitative, descriptive, cross-sectional design to assess the nutritional status of Grade seven adolescents in urban Tshwane. This methodological framework combined a survey-based approach for gathering socioeconomic data and a standardised observational study based on a standardised anthropometric measurement protocol. By using these two data collection streams, the researchers systematically calculated (BMI-for-age) z-scores and waist-to-height ratios (WHtR) according to WHO standards. This operational structure helped to provide a strong measure of both general and central adiposity while measuring family affluence using validated indicators.

### Population and sampling

The study employed a stratified random sampling approach to select five public primary schools in urban Tshwane, ensuring representation across socioeconomic quintiles four and five (Van Dyk & White 2019). Convenience sampling was employed to sample the respondents. The sample was derived from a total district sample frame of 20 008 Grade seven pupils. The initial target sample size was 200 adolescents (40 per school), calculated to achieve sufficient precision in estimating the prevalence of overweight and obesity. The sample size was determined using the standard formula for prevalence studies (Naing, Winn & Nordin 2006):


$$\text{Sample size}=Z^{2}*p*(1-p)/c^{2}$$

where

- *Z* = 1.96 (for 95% confidence level),
- *p* = 0.8 (anticipated prevalence based on prior South African studies; Otitoola et al. 2020),
- *c* = 0.065 (6.5% margin of error).

This calculation yielded 189 respondents, rounded to 200 to account for non-response. The final sample included 164 adolescents (86 girls, 78 boys), aged 11–14 years, achieving an 82% response rate. Respondents were recruited from five schools: H2 (*n* = 23), A5 (*n* = 38), E4 (*n* = 43), P3 (*n* = 21) and S1 (*n* = 39). Stratification by school ensured socioeconomic diversity, with fee-paying schools (quintiles 4–5) reflecting Tshwane’s urban obesogenic environment.

The Department of Basic Education (DBE) restricted access during teaching contact times; therefore, arrangements were confirmed with all schools to engage participants on the conclusion of second-term assessments. However, students are not required to be at school once assessments have concluded. This resulted in fewer participants being available and necessitated rescheduling the research engagement at the selected schools for the commencement of the third term in 2024.

### Data collection and instrumentation

The research employed a composite questionnaire containing socio-demographic questions (age, sex and school) and the validated Family Affluence Scale (FAS) (Corell et al. 2021; Hartley, Levin & Currie 2016) to assess socioeconomic status (SES), alongside a comprehensive survey to anthropometric measurements. Research personnel consisted of registered dietitians and nutritionists from a Tshwane-based university who underwent rigorous training on standardised measurement protocols and ethical procedures during two half-day sessions. The FAS was used to measure SES through its validated items, which included household car ownership and bedroom occupancy and digital device ownership (number of laptops, cell phones and television sets) and family holiday frequency. The cumulative FAS score was used to classify participants into low or middle affluence categories, as per established guidelines. The study also included a self-reported income as part of socioeconomic measurement.

The anthropometric assessments followed WHO standardised procedures (Gibson 2024; WHO 2007) where height was measured to the nearest 0.1 cm using a Seca stadiometer and weight recorded to the nearest 0.1 kg using a calibrated Philips electronic scale (Model HF350). The anthropometric measurements were conducted twice with the final mean value used for analysis (Gibson 2024). The BMI calculation formula according to the standard method is BMI = weight (kg)/ height (m^2^). The waist circumference measurement followed WHO protocols (WHO 2011) by taking the midpoint between the iliac crest and lower rib margin, while participants stood erect after normal expiration. The WHtR was derived using the formula: WHtR = waist circumference (cm)/height (cm). The analytical approach incorporated quality control measures, including duplicate measurements and standardised data collection protocols to minimise inter-observer variability. The research protocol used WHO-standardised anthropometric assessments (WHO 2007, 2011) together with validated socioeconomic indicators (Hartley et al. 2016). These instruments provided a robust framework for accurately assessing the nutritional status of the adolescent cohort. Overall, the methodology has been previously validated in similar epidemiological studies of paediatric obesity (Gibson 2024; Khara et al. 2023).

### Data analysis

The FAS was used to assess family affluence through its validated multiple household asset indicators. Each item was scored and summed to yield a composite affluence score, which was then categorised into ‘low’ and ‘middle’ affluence groups. Furthermore, the research analysed socioeconomic variables through ordinal measurement by evaluating household income brackets and parental education levels as potential determinants of nutritional status in line with South African youth health studies (Ntimana et al. 2024). Anthropometric data were analysed using the WHO (2007) growth reference standards. Body mass index z-scores were categorised as underweight (< −2 standard deviation [s.d.]), normal weight (−2 to +1 s.d.), overweight (> +1 to +2 s.d.) and obese (> +2 s.d.). The WHtR values were calculated and categorised based on established cardiometabolic risk criteria: WHtR < 0.5 was classified as low risk, WHtR ≥ 0.5 was classified as increased cardiometabolic risk and WHtR ≥ 0.6 was classified as substantially increased risk (Ashwell & Gibson 2016). The established criteria were used to interpret these indices where WHtR values above the 90th percentile indicated elevated cardiometabolic risk (Seo & Shim 2019).

### Statistical analysis

The research study applied a strict methodological framework together with specialised software tools for statistical analysis. The data processing and statistical computations were performed using IBM SPSS Statistics version 29 (IBM Corp., 2022, IBM SPSS Statistics for Windows, Version 29.0, IBM, Armonk, NY), which enabled comprehensive descriptive analyses, including calculation of means, standard deviations and frequency distributions for anthropometric variables (George & Mallery 2024). The software enabled researchers to perform complex analytical procedures through cross-tabulations with chi-square tests for studying relationships between socioeconomic indicators and nutritional status according to modern nutritional epidemiology standards (Coenen, Batterham & Beck 2021). For growth standard classifications, the study utilised WHO AnthroPlus software (the World Health Organisation, Geneva, Switzerland), which applies the WHO (2007) growth references to calculate BMI-for-age z-scores, ensuring international comparability of findings (De Onis et al. 2012). The dual-software system allowed researchers to study both continuous variables (height, weight and waist circumference) and categorical variables (BMI categories and WHtR risk classifications) in a manner that was consistent with current paediatric obesity research methods (Ntimana et al. 2024).

### Validity and reliability

The validity of the study was ensured with the use of standardised and internationally recognised methods of measurement. Anthropometric measurements were performed following the protocols of the WHO, and BMI-for-age z-scores were obtained with the WHO growth reference standards, which ensured that the adiposity of the adolescents was accurately represented. The inclusion of both BMI and waist-to-height ratio increases construct validity as it gives an overall picture of general and central adiposity. In addition, SES was assessed with the validated FAS and enhances the validity of instruments based on their use in adolescent health research. While stratified sampling across five schools provides for enhanced representativeness in the urban Tshwane context, external validity is somewhat diminished because the focus was on fee-paying schools, which may not reflect the broader socioeconomic spectrum. Reliability was assured by careful data collection procedures and quality controls. Anthropometric measurements were carried out by trained persons using calibrated instruments, each measurement being repeated twice and the average measurement being taken to reduce random error and to increase consistency. Use of standardised software such as WHO AnthroPlus and SPPS further contributes to analytical reliability through the standardisation of classification and statistical processing. However, there are still some limitations, and they come particularly in the fact that the study relied on self-reported socioeconomic data, which could possibly introduce recall or social desirability bias, as well as the convenience sampling at the participant level that may affect the consistency of the findings in different samples.

### Ethical considerations

Ethics approval was granted by the University of the Free State Ethics Committee (UFS-‘HSD2024/0150’). Written permission to work with the Tshwane primary schools was obtained from the Gauteng DBE (2024/22) and heads of the participating schools. Written informed consent from parents and guardians of the students was obtained for their children’s participation in the study, and written assent for each child participant was obtained. To ensure confidentiality, all data were anonymised by assigning unique participant identification codes in order to prevent any linkage of personally identifiable information to the research records. Electronic data were stored on password-protected computers, while hard copy documents, such as consent and assent forms, were kept in a locked filing cabinet located in a restricted access. In accordance with institutional research data management policies, all research data will be securely stored for a period of 5 years after the study is completed, after which electronic files will be permanently deleted and physical documents will be shredded.

## Results

### Sociodemographic characteristics

Table 1a and Table 1b presents the sociodemographic characteristics of the study sample, which consisted of 164 early adolescents drawn from five public primary schools.

**TABLE 1a:** Sociodemographic characteristics.

Gender	Age (years)				Total (*n*)
	11	12	13	14	
Girls	2	48	33	3	86
Boys	0	42	33	3	78

**Total**	**2**	**90**	**66**	**6**	**164**

**TABLE 1b:** Sociodemographic characteristics.

Socioeconomic indicator	Level	*n*	%
**Affluence level**	Low affluence	112	68.3
	Middle affluence	52	31.7
**Mother’s formal education level**	Grade 4–8	1	0.6
	Grade 9–12	34	20.7
	Undergraduate	48	29.3
	Post-graduate	69	42.1
	Don’t know	6	3.7
**Father’s formal education level**	None	2	1.2
	Grade 9–12	24	14.6
	Degree or diploma	37	22.6
	Post-graduate qualification	68	41.5
	Don’t know	29	17.7

The age and gender distribution indicates a relatively balanced sample, with 86 boys (52.4%) and 78 girls (47.6%). Participants were predominantly concentrated in the 12- and 13-year age brackets, which together accounted for 95.1% of the total sample. Specifically, 54.9% (*n* = 90) of the participants were aged 12 years, and 40.2% (*n* = 66) were aged 13 years. Very few respondents were aged 11 (*n* = 2; 1.2%) or 14 (*n* = 6; 3.7%). Boys were slightly more represented across all age categories except age 13, where representation was equal between genders (*n* = 33 per group). The age distribution reflects a relatively proportional participation of boys and girls. In terms of household affluence, a substantial majority of respondents (*n* = 112; 68.3%) were categorised as living in low-affluence households, while only 31.7% (*n* = 52) were identified as having middle-level affluence. Among mothers, educational attainment was skewed towards higher education, with 29.3% (*n* = 48) holding undergraduate degrees and 42.1% (*n* = 69) possessing postgraduate qualifications. A small proportion of respondents (3.7%) reported not knowing their mother’s level of education. Similarly, fathers’ educational attainment showed a high prevalence of tertiary education. Specifically, 22.6% (*n* = 37) of fathers held undergraduate degrees, and 41.5% (*n* = 68) had postgraduate qualifications. However, a noteworthy 17.7% (*n* = 29) of respondents reported not knowing their father’s educational level, which may reflect the absence of paternal figures in the household or limited communication regarding paternal background.

### Obesity prevalence by gender

Table 2 summarises the BMI-for-age z-score distribution by gender and age group using the WHO 2007 reference. Confidence intervals (CI) are provided for selected categories. Overweight is defined as > +2 s.d. and obesity as > +3 s.d.

**TABLE 2:** Summary of height-for-age z-score and (body mass index-for-age z-score) by sex and age with 95% confidence intervals.

Sex	Age	*N*	< −2 s.d. BAZ (%) (95% CI)	< −2 s.d. HAZ (%) (95% CI)	> +1 s.d. BAZ (%) (95% CI)	> +2 s.d. BAZ (%) (95% CI)	> +3 s.d. BAZ (%) (95% CI)	Mean BAZ ± s.d.	Mean HAZ ± s.d.
Girls	12	22	0.0 (0.0–0.0)	4.5 (0.0–13.2)	54.5 (33.7–75.4)	36.4 (16.3–56.5)	4.5 (0.0–13.2)	1.02 ± 1.60	0.25 ± 2.04
	13	53	1.9 (0.0–5.5)	7.5 (0.4–14.7)	43.4 (30.1–56.7)	24.5 (12.9–36.1)	13.2 (4.1–22.3)	1.05 ± 2.16	−0.39 ± 2.39
	14	11	9.1 (0.0–26.1)	9.1 (0.0–26.1)	18.2 (0.0–41.0)	9.1 (0.0–26.1)	0.0 (0.0–0.0)	−0.23 ± 1.52	−0.51 ± 1.34
Boys	12	18	5.6 (0.0–16.1)	11.1 (0.0–25.6)	50.0 (26.9–73.1)	16.7 (0.0–33.9)	5.6 (0.0–16.1)	0.82 ± 1.54	0.53 ± 2.75
	13	52	3.8 (0.0–9.1)	3.8 (0.0–9.1)	30.8 (18.2–43.3)	15.4 (5.6–25.2)	7.7 (0.4–14.9)	0.60 ± 1.73	0.23 ± 1.64
	14	8	0.0 (0.0–0.0)	0.0 (0.0–0.0)	25.0 (0.0–55.0)	0.0 (0.0–0.0)	0.0 (0.0–0.0)	−0.15 ± 1.24	−0.65 ± 0.99

HAZ, height-for-age z-score; BMI, body mass index; BAZ, BMI-for-Age z-score; s.d., standard deviation; CI, confidence intervals.

Across the total sample, the prevalence of thinness (BAZ < −2 s.d.) remained low across all age and sex strata, ranging from 0.0% to 9.1%. Notably, thinness was absent among 12-year-old girls and minimal among boys of the same age (5.6%; 95% CI: 0.0–16.1). The highest thinness prevalence was observed among 14-year-old girls (9.1%; 95% CI: 0.0–26.1), though the wide CI reflects the small sample size (*n* = 11). In contrast, stunting (HAZ < −2 s.d.), which is indicative of chronic undernutrition, was more prevalent, particularly among boys aged 12 (11.1%; 95% CI: 0.0–25.6) and girls aged 14 years (9.1%; 95% CI: 0.0–26.1). Girls aged 13 showed a stunting prevalence of 7.5% (95% CI: 0.4–14.7), while 13-year-old boys had a lower rate at 3.8% (95% CI: 0.0–9.1). The prevalence of overnutrition, which is defined by BAZ > +1 s.d., was substantially higher. Among girls, 54.5% of 12-year-olds (95% CI: 33.7–75.4) and 43.4% of 13-year-olds (95% CI: 30.1–56.7) were classified as at risk of overweight. The prevalence declined among 14-year-old girls to 18.2% (95% CI: 0.0–41.0). A similar pattern was observed among boys, with 50.0% of 12 year olds (95% CI: 26.9–73.1) and 30.8% of 13 year olds (95% CI: 18.2–43.3) at risk. More critically, overweight and obesity (BAZ > +2 s.d.) affected 36.4% (95% CI: 16.3–56.5) of 12-year-old girls and 24.5% (95% CI: 12.9–36.1) of 13-year-old girls. Among boys, 16.7% (12 years) and 15.4% (13 years) were overweight. The obesity burden (BAZ > +3 s.d.), though lower, was non-negligible. It peaked at 13.2% (95% CI: 4.1–22.3) among 13-year-old girls and 7.7% (95% CI: 0.4–14.9) among 13-year-old boys. The mean BAZ scores for most age-sex groups were positive and ranged from 0.60 ± 1.73 among 13-year-old boys to 1.05 ± 2.16 among 13-year-old girls, signifying a general trend towards overweight. The corresponding HAZ values ranged from −0.51 ± 1.34 (14-year-old girls) to +0.53 ± 2.75 (12-year-old boys), suggesting heterogeneity in linear growth outcomes possibly related to early childhood nutritional and health conditions.

### Waist-to-height ratio results

**FIGURE 1:**
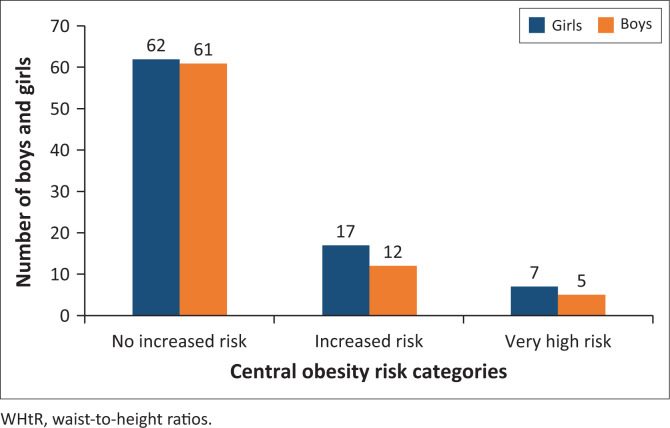
Waist-to-height ratio by gender.

Figure 1 presents the distribution of WHtR risk categories stratified by gender, offering insight into the differential cardiometabolic risk profiles among boy and girl participants.

The data reveal that the majority of individuals, irrespective of gender, fall within the ‘No increased risk’ category, with 62 girls and 61 boys classified as such. This suggests that a substantial proportion of both sexes exhibit WHtR values below the established risk threshold (typically < 0.5), indicating a generally favourable adiposity profile in terms of central fat distribution for most participants.

However, closer scrutiny of the elevated risk categories reveals a noteworthy gender disparity. Specifically, a higher number of girls (17) than boys (12) fall into the ‘Increased risk’ category, suggesting that a greater proportion of girls may be exhibiting central adiposity that approaches clinically concerning thresholds. This trend is further reinforced in the ‘Very high risk’ category, where seven girls compared to five boys were identified.

### Association between socioeconomic status and nutritional status

Table 3 presents the results of chi-square analyses examining the distribution of BMI and WHtR categories across selected socioeconomic variables, namely parental education levels, household income and perceived affluence. These results offer insights into the complex interrelationship between SES and adolescent adiposity patterns, interpreted through both general obesity classification (BMI) and central adiposity risk (WHtR).

**TABLE 3:** Distribution of body mass index and waist-to-height ratio categories by socioeconomic variables.

Socioeconomic factors	Variable	BMI categories				WHtR_categories			*p*-value	
		Underweight	Normal	Overweight	Obese	No Increased Risk	Increased Risk	Very High Risk	(BMI)	(WHtR)
The mother’s highest Education level	Don’t know	0	2	1	1	2	1	1	0.057	0.162
	Grade 4–8	0	0	0	1	0	1	0	-	-
	Grade 9–12	13	9	6	5	19	10	4	-	-
	Post-graduate	29	31	2	6	53	10	5	-	-
	Undergraduate	17	19	6	4	36	8	2	-	-
The father’s highest formal education level	Don’t know	10	12	4	0	19	6	1	0.361	0.488
	Grade 9–12	7	9	2	6	13	8	3	-	-
	None	0	2	0	0	1	1	0	-	-
	Post-graduate	29	23	5	7	50	9	5	-	-
	Undergraduate	13	15	4	4	27	6	3	-	-
Household income	Above R20 000	32	36	10	11	64	18	7	0.043†	0.004
	R10 001–R20 000	18	20	4	1	33	9	1	-	-
	R1001–R3500	2	2	0	0	4	0	0	-	-
	R3501–R5000	1	0	1	3	1	1	3	-	-
	R5001–R10 000	6	3	0	2	8	2	1	-	-
Affluence	Low	39	37	12	15	70	22	11	0.123	0.109
	Middle	20	24	3	2	40	8	1	-	-

BMI, body mass index; WHtR, waist-to-height ratios.

† , Significant at the 0.05 level (two tailed).

Although the associations between BMI categories and both maternal and paternal education levels were not statistically significant at the conventional threshold (*p* = 0.057 for mothers; *p* = 0.361 for fathers), some notable patterns emerge. Adolescents whose mothers had postgraduate level education exhibited a predominance in the ‘normal’ BMI category (*n* = 31) and the ‘no increased risk’ WHtR category (*n* = 53), suggesting a protective effect of higher maternal education on overall and central adiposity. Similarly, fathers with postgraduate qualifications were associated with the largest counts in the ‘normal’ BMI (*n* = 23) and ‘no increased risk’ WHtR categories (*n* = 50). Conversely, lower parental educational attainment, particularly among those reporting ‘Don’t know’ or only primary schooling, was associated with a more diffuse spread across higher-risk categories although small cell sizes in these groups warrant cautious interpretation. While these trends are suggestive, the absence of statistically significant associations implies that education alone may not independently predict adolescent weight status in this context, or that the sample size limits power to detect modest effects. In contrast, household income demonstrated a statistically significant association with both BMI (*p* = 0.043) and WHtR (*p* = 0.004) categories, indicating a more robust and consistent relationship between economic resources and adiposity. Adolescents from the highest income bracket (above R20 000 per month) were more likely to fall into the ‘normal’ BMI category (*n* = 36) and ‘no increased risk’ WHtR group (*n* = 64). This trend suggests that greater household wealth may afford dietary quality, healthcare access and supportive living environments conducive to healthier growth trajectories. Conversely, lower income groups, particularly those earning between R1001–R3500 or R3501–R5000, had a disproportionate number of adolescents in the overweight or obese and higher WHtR risk categories. For instance, adolescents in the R3501–R5000 category, though small in number, included three cases classified as ‘very high risk’ by WHtR, a striking concentration of central adiposity risk within a low-income subgroup. Perceived affluence did not demonstrate a statistically significant association with either BMI (*p* = 0.123) or WHtR (*p* = 0.109), though the distribution patterns remain informative. The low affluence group encompassed the largest absolute number of adolescents in both ‘overweight’ (*n* = 12) and ‘obese’ (*n* = 15) BMI categories, as well as a disproportionately high number in the ‘very high risk’ WHtR group (*n* = 11). By contrast, adolescents from middle-affluence households were more concentrated in the ‘normal’ BMI and ‘no increased risk’ WHtR categories, indicating a potentially protective role of moderate socioeconomic standing.

## Discussion

This study provides further compelling evidence of the rising burden of overweight and obesity among urban South African adolescents, particularly among girls. It is consistent with broader epidemiological trends across LMICs undergoing rapid nutrition transition (Popkin & Ng 2022) while identifying important local specifics that ought to be addressed in public health planning. The clear gender difference found in this study is reflective of a well known but poorly understood trend in nutritional epidemiology. Recent multi-country analyses from the NCD Risk Factor Collaboration (2024) indicate that urban African adolescent girls experience especially high excess adiposity compared with boys, notably, the higher burden symptomised by those with early onset of overweight, obesity or abdominal obesity. This pattern may reflect the interaction of biological factors (e.g. earlier puberty), participation in physical activity limited by socio-cultural expectations (Ntimana et al. 2024) and gendered dietary differences (Lombardo et al. 2024). These results emphasise the vulnerability of early adolescent girls, which is noteworthy given that this developmental window is characterised by the establishment of lifestyle habits that may track into adulthood (Patton et al. 2016). The evidence for the value of including biomarkers of central adiposity is strongly supported by the result of this study although the extent to which biomarkers are systematically applicable from an epidemiological point of view remains to be further evaluated. The large number of adolescents with elevated cardiometabolic risk identified by use of WHtR strengthens rising concerns over the limited applicability of BMI alone as a population screening tool in African populations. Recent studies of body composition indicate that global BMI references may not accurately predict metabolic risk in African adolescents (Ntimana et al. 2024; Tydeman-Edwards, Van Rooyen & Walsh 2018). These youth often maintain a more favourable metabolic profile at BMI levels where global standards would typically predict the onset of obesity-related complications. This has clinical implications while suggesting that existing screening approaches may fail to identify risk in this population. Socioeconomic patterning of obesity risk in this study is particularly complex in view of South Africa’s earlier stages of nutrition transition. While earlier nutrition transition theory associated higher household income with the highest obesity risk, this study finds a narrowing gap. Contrary to expectations, there were no clear educational gradients across strata, suggesting that the urban obesogenic environment in Tshwane affects adolescents across the socioeconomic spectrum. This helps to contest simple assumptions over the socioeconomic determinants of obesity. Recent research on food environments in urban South Africa (Phetla & Skaal 2023; Pineda et al. 2024) helps to explain this by showing how pervasive use of junk food marketing, limited availability of affordable healthy alternatives and sedentary urban lifestyles create obesogenic environments that impact all socioeconomic groups but through different pathways. Coexistence of under- and overnutrition among this study population marks the ‘double burden of malnutrition’ that has increasingly typified urban Africa (Popkin 2001). Documented in several recent studies (Hall et al. 2024; Tan et al. 2024), this poses a significant challenge for health systems predominantly geared to address either underweight or overweight but not both. Biological consequences of this dual burden may be particularly stark, as indicated by emerging investigations into the developmental origins of health and disease in South African cohorts (Davies et al. 2018).

### Strength and limitations

Methodologically, this study contributes to ongoing debate over optimal approaches to nutritional surveillance among adolescents. Complementary use of BMI and WHtR is consistent for metabolic risk assessment while also highlighting the need for further research to ascertain appropriate cut-off points for African populations (Lo et al. 2016; Sweatt, Garvey & Martins 2024). Agreement of our findings with recent longitudinal studies (University of Cape Town & University of Michigan [CAPS] 2012) strengthens the robustness of our finding patterns and underlines their likely persistence into adulthood. Furthermore, the study has important methodological strengths that enhance the validity and applicability of its findings. Initially, the use of standardised protocols of the WHO for anthropometric measures that were performed by trained staff assures the reliability of the main outcome variables. Inclusion of BMI and WHtR as complementary measures allows for a more complete assessment of adiposity and metabolic risk than what each single measure can achieve in isolation, in alignment with current best practices in paediatric obesity research (Ntimana et al. 2024; Sweatt et al. 2024). The stratified random sampling across different schools in urban Tshwane lent socioeconomic, geographic and educational diversity, rendering the sample representative of the target population. This methodological rigour is particularly relevant in view of the scarcity of localised data on obesity among adolescents in South African urban environments (SANHANES-2 2024). The study’s focus on early adolescence (Grade 7) allows investigation of a crucial window for obesity prevention, which includes a transition period in the evolution of health behaviour in the life course (Patton et al. 2016). Inclusion of multiple socioeconomic indicators (household income, parental education and family affluence) allowed for a more differentiated view on putative determinants, going beyond simply unidimensional assessments as have been the norm in other studies. This is particularly relevant for socio-economic transitions in the South African context (Ntimana et al. 2024). Several limitations should be considered when interpreting the results of the study. The cross-sectional design does not allow for causal inference on the links between the factors that we observed and obesity outcomes. Although the sample size allowed for adequate prevalence estimates, it may have been underpowered to detect more subtle associations, particularly in subgroup analyses. This is indicated by wide CI around certain estimates, which is a common challenge for studies in this context that use schools as sampling frames because of logistical constraints (Setia 2016). Given that our sample included mostly English-medium fee-paying schools, notwithstanding the observed variation in obesity between communities, caution is warranted before generalising the findings to non-fee-paying schools and all other socioeconomic strata. Recent evidence points out that the patterns for obesity may vary substantially between school types in South Africa (Nyawose et al. 2024). We relied on self-report measures of socioeconomic data, which may be affected by recall and social desirability biases. Less subjective measures of household circumstances may have been more revealing (Pineda et al. 2024). Lack of data on eating habits and physical activity limits interpretation through understanding of putative mediators of behaviour in association with the phenomenon, which is known as an important area for future research (Lombardo et al. 2024). While this study provides important local evidence, the single-city design suggests that caution is warranted in extrapolating the findings to other urban settings in South Africa and the region, given the marked variation in food environment and conditions of urban infrastructure (Phetla & Skaal 2023), as well as regional variation in levels of overweight and obesity. Anthropometric measures alone are insufficient to capture all health risks (Goedecke et al. 2022).

## Conclusion

This research underscores the concerning levels of overweight, obesity and central adiposity among young adolescents residing in urban Tshwane – one of the burgeoning public health concerns in South Africa’s rapidly urbanising environments that is often overlooked. These burdens disproportionately affected adolescent girls, echoing emerging regional and global trends and highlighting gender as a relevant axis of vulnerability to nutritional risk. The persistent prevalence of stunting observed, particularly among younger adolescents, signals the lingering presence of early-life nutritional deficiencies, warranting ongoing surveillance. In coexistence with overweight and obesity, these findings reinforce the notion of a DBM, further indicating a transitionary nutritional landscape that defies a simple chronic-overweight binary and calls for dual-track interventions. The implications of these findings for policy are clear. Gender-sensitive interventions to meet the specific vulnerabilities of adolescent girls are critically needed. Public health screening must move beyond simple BMI indicators to incorporate additional markers, such as waist-to-height ratio, for legible identification of cardiometabolic risk. Effective prevention efforts against obesity will require broad-based approaches that simultaneously address food environments, urban design and socioeconomic inequalities. Evidence from similar settings suggests the most effective approaches include combinations of school-based interventions with food environment regulations and community engagement. Further research should prioritise longitudinal designs tracking the transition of obesity from adolescence into adulthood and mixed methods that capture the relative contributions of the biological, behavioural and environmental in determining risk. Further development and validation of population-specific screening tools, particularly contextually relevant waist-to-height ratio cut-points, should be prioritised to ensure appropriate identification of risk in African populations. While descriptive investigations suggested associations between some socioeconomic variables, particularly household income, and increases in adiposity, statistical significance was not always detected. This apparent paradox may reflect the complexity of nutrition transitions in urban low- and middle-income settings, where obesogenic environments are ubiquitous across all socioeconomic groups but through varying pathways. The absence of a socio-economic gradient further reinforces the need for a socioecological framework that encompasses the interplay between biological, behavioural and environmental levels. The public health implications are clear. Adolescents in urban South Africa face increasing exposure to both structural and behavioural risk factors that, if left unaddressed, will fuel the early onset of NCDs, adding strain to an already burdened health system. Multisectoral interventions that are gender responsive, equity-oriented and contextually embedded are urgently required. Policy initiatives should prioritise transforming food environments and strengthening school-based physical activity while institutionalising screening and prevention programmes specific to adolescents. Only through integrative, evidence-based and context-specific approaches can the growing risk of adolescent obesity be mitigated, allowing South Africa to secure its long-term health capital among the youth.

## References

[CIT0001] Alberga, A.S., Sigal, R.J., Goldfield, G., Prud’homme, D. & Kenny, G.P., 2012, ‘Overweight and obese teenagers: Why is adolescence a critical period?’, *Pediatric Obesity* 7(4), 261–273. 10.1111/j.2047-6310.2011.00046.x 22461384

[CIT0002] Ashwell, M. & Gibson, S., 2016, ‘Waist-to-height ratio as an indicator of ‘early health risk’: Simpler and more predictive than using a ‘matrix’ based on BMI and waist circumference’, *BMJ Open* 6(3), e010159. 10.1136/bmjopen-2015-010159 PMC4800150 26975935

[CIT0003] Coenen, A., Batterham, M.J. & Beck, E.J., 2021, ‘Statistical methods and software used in nutrition and dietetics research: A review of the published literature using text mining’, *Nutrition & Dietetics* 78(3), 333–342. 10.1111/1747-0080.12678 34155748 PMC8362035

[CIT0004] Corell, M., Chen, Y., Friberg, P., Petzold, M. & Löfstedt, P., 2021, ‘Does the family affluence scale reflect actual parental earned income, level of education and occupational status? A validation study using register data in Sweden’, *BMC public health* 21(1), 1995. 10.1186/s12889-021-11968-2 34732163 PMC8565642

[CIT0005] Davies, J.I., Macnab, A.J., Byass, P., Norris, S.A., Nyirenda, M., Singhal, A. et al., 2018, Developmental origins of health and disease in Africa – Influencing early life, *The Lancet Global Health* 6(3), e244–e245. 10.1016/S2214-109X(18)30036-6 29433658

[CIT0006] De Amicis, R., Mambrini, S.P., Pellizzari, M., Foppiani, A., Bertoli, S., Battezzati, A. et al., 2022, ‘Ultra-processed foods and obesity and adiposity parameters among children and adolescents: A systematic review’, *European Journal of Nutrition* 61(5), 2297–2311. 10.1007/s00394-022-02873-4 35322333 PMC8942762

[CIT0007] De Onis, M., Onyango, A., Borghi, E., Siyam, A., Blössner, M., Lutter, C. et al., 2012. ‘Worldwide implementation of the WHO child growth standards’, *Public Health Nutrition* 15(9), 1603–1610. 10.1017/S136898001200105X 22717390

[CIT0008] George, D. & Mallery, P., 2024, *IBM SPSS statistics 29 step by step: A simple guide and reference*, Routledge, London.

[CIT0009] Gibson, R.S., 2024, *Principles of nutritional assessment*, 3rd edn., viewed 2 February 2026, from https://nutritionalassessment.org/.

[CIT0010] Goedecke, J.H., Nguyen, K.A., Kufe, C., Masemola, M., Chikowore, T., Mendham, A.E. et al., 2022, ‘Waist circumference thresholds predicting incident dysglycaemia and type 2 diabetes in Black African men and women’, *Diabetes Obesity Metabolism* 24(5), 918–927. 10.1111/dom.14655 35088498 PMC9305761

[CIT0011] Hall, K., Almeleh, C., Giese, S., Mphaphuli, E., Slemming, W., Mathys, R. et al., 2024, *South African early childhood review 2024*, Children’s Institute, University of Cape Town and Ilifa Labantwana, Cape Town.

[CIT0012] Hartley, J.E., Levin, K. & Currie, C., 2016, ‘A new version of the HBSC Family Affluence Scale – FAS III: Scottish qualitative findings from the international FAS development study’, *Child Indicators Research* 9, 233–245. 10.1007/s12187-015-9325-3 26925177 PMC4757604

[CIT0013] Khara, T., Myatt, M., Sadler, K., Bahwere, P., Berkley, J.A., Black, R.E. et al., 2023, ‘Anthropometric criteria for best-identifying children at high risk of mortality: A pooled analysis of twelve cohorts’, *Public Health Nutrition* 26(4), 803–819. 10.1017/S136898002300023X 36734049 PMC10131149

[CIT0014] Kuzik, N., Da Costa, B.G.G., Hwang, Y., Verswijveren, S.J.J.M., Scott Rollo, S., Tremblay, M.S. et al., 2022, ‘School-related sedentary behaviours and indicators of health and well-being among children and youth: A systematic review’, *International Journal of Behavioral Nutrition and Physical Activity* 19, 40. 10.1186/s12966-022-01258-4 35382825 PMC8979786

[CIT0015] Lo, K., Wong, M., Khalechelvam, P. & Tam, W., 2016, ‘Waist-to-height ratio, body mass index and waist circumference for screening paediatric cardio-metabolic risk factors: A meta-analysis’, *Obesity Reviews* 17(12), 1258–1275. 10.1111/obr.12456 27452904

[CIT0016] Lombardo, M., Feraco, A., Armani, A., Camajani, E., Gorini, S., Strollo, R. et al., 2024, ‘Gender differences in body composition, dietary patterns, and physical activity: Insights from a cross-sectional study’, *Frontiers in Nutrition* 11, 1414217. 10.3389/fnut.2024.1414217 39055386 PMC11271261

[CIT0017] Malik, V.S. & Hu, F.B., 2022, ‘The role of sugar-sweetened beverages in the global epidemics of obesity and chronic diseases’, *Nature Reviews Endocrinology* 18(4), 205–218. 10.1038/s41574-021-00627-6 PMC8778490 35064240

[CIT0018] Naicker, N., Mathee, A. & Teare, J., 2015, ‘Food insecurity in households in informal settlements in urban South Africa’, *South African Medical Journal* 105(4), 268–270. 10.7196/samj.8927 26294864

[CIT0019] Naing, L., Winn, T. & Nordin, R., 2006, ‘Practical issues in calculating the sample size for prevalence studies’, *Archives of Orofacial Sciences* 1, 9–14.

[CIT0020] NCD Risk Factor Collaboration (NCD-RisC), 2024, ‘Worldwide trends in underweight and obesity from 1990 to 2022: A pooled analysis of 3663 population-representative studies with 222 million children, adolescents, and adults’, *The Lancet* 403(10431), 1027–1050. 10.1016/S0140-6736(23)02750-2 PMC7615769 38432237

[CIT0021] Ngwenya, N.A. & Ramukumba, T.S., 2017, ‘Prevalence of adolescent obesity at a high school in the city of Tshwane’, *Curationis* 40(1), e1662. 10.4102/curationis.v40i1.1662 PMC6091632 28582980

[CIT0022] Ntimana, C.B., Seakamela, K.P., Mashaba, R.G. & Maimela, E., 2024, ‘Determinants of central obesity in children and adolescents and associated complications in South Africa: A systematic review’, *Frontiers in Public Health* 12, 1324855. 10.3389/fpubh.2024.1324855 38716247 PMC11075369

[CIT0023] Nyawose, Z.Z., Naidoo, R., Christie, C., Bassett, S., Coetzee, D., Van Gent, M. et al., 2024, ‘Results from South Africa’s 2022 healthy active kids’ report card on physical activity, body composition proxies, and nutritional status in children and adolescents’, *Journal of Physical Activity and Health* 21(9), 861–871. 10.1123/jpah.2023-0708 39117305

[CIT0024] Okop, K.J., Agabi, Y.A. & Joseph, V., 2025, ‘Weight underestimation and high cardiovascular disease risk among South African adults with obesity: Implications for integrated obesity prevention’, *BMC Public Health* 25, 2087. 10.1186/s12889-025-23378-9 40468282 PMC12139319

[CIT0025] Onyango, A.W., Jean-Baptiste, J., Samburu, B. & Mahlangu, T.L.M., 2019, ‘Regional overview on the double burden of malnutrition and examples of program and policy responses: African region’, *Annals of Nutrition & Metabolism* 75(2), 127–130. 10.1159/000503671 31743899

[CIT0026] Otitoola, O., Oldewage-Theron, W. & Egal, A., 2020, ‘Prevalence of overweight and obesity among selected schoolchildren and adolescents in Cofimvaba, South Africa’, *South African Journal of Clinical Nutrition* 34(3), 97–102. 10.1080/16070658.2020.1733305

[CIT0027] Patton, G.C., Sawyer, S.M., Santelli, J.S., Ross, D.A., Afifi, R., Allen, N.B. et al., 2016, ‘Our future: A Lancet commission on adolescent health and wellbeing’, *The Lancet* 387(10036), 2423–2478. 10.1016/S0140-6736(16)00579-1 PMC5832967 27174304

[CIT0028] Phetla, M.C. & Skaal, L., 2023, ‘Scanning for obesogenicity of primary school environments in Tshwane, Gauteng, South Africa’, *International Journal of Environmental Research and Public Health* 20(19), 6889. 10.3390/ijerph20196889 37835158 PMC10572655

[CIT0029] Phetla, M.C., Skaal, L. & Chelule, K.P., 2024, ‘Dietary habits among primary school learners in the Tshwane West District of Gauteng South Africa’, *Health SA Gesondheid* 29, 2746. 10.4102/hsag.v29i0.2746 39649351 PMC11621916

[CIT0030] Pineda, E., Stockton, J., Scholes, S., Lassale, C. & Mindell, J.S., 2024, ‘Food environment and obesity: A systematic review and meta-analysis’, *BMJ Nutrition, Prevention & Health* 7(1), 204–211. 10.1136/bmjnph-2023-000663 PMC11221287 38966119

[CIT0031] Popkin, B.M., 2001, ‘The nutrition transition and obesity in the developing world’, *The Journal of Nutrition* 131(3), 871S–873S. 10.1093/jn/131.3.871S 11238777

[CIT0032] Popkin, B.M. & Ng, S.W., 2022, ‘The nutrition transition to a stage of high obesity and noncommunicable disease prevalence dominated by ultra-processed foods is not inevitable’, *Obesity Reviews* 23(1), e13366. 10.1111/obr.13366 34632692 PMC8639733

[CIT0033] SANHANES-2, 2024, *The second South African national health and nutrition examination survey (SANHANES-2): National results*, HSRC Press, Cape Town.

[CIT0034] Seo, S.H. & Shim, Y.S., 2019, ‘Association of sleep duration with obesity and cardiometabolic risk factors in children and adolescents: A population-based study’, *Scientific Reports* 9, 9463. 10.1038/s41598-019-45951-0 31263172 PMC6603036

[CIT0035] Setia, M.S., 2016, ‘Methodology series module 3: Cross-sectional studies’, *Indian Journal of Dermatology* 61(3), 261–264. 10.4103/0019-5154.182410 27293245 PMC4885177

[CIT0036] Shisana, O., Labadarios, D., Rehle, T., Simbayi, L., Zuma, K., Dhansay, A. et al., 2013, *South African National Health and Nutrition Examination Survey (SANHANES-1)*, HSRC Press, Cape Town.

[CIT0037] Shrimpton, R. & Rokx, C., 2012, *The double burden of malnutrition: A review of global evidence*, World Bank, Washington, DC.

[CIT0038] Statistics South Africa, 2024, *General Household Survey 2023* (Statistical release P0318), viewed 23 March 2025, from https://www.statssa.gov.za/?page_id=1854&PPN=P0318&SCH=73897.

[CIT0039] Sweatt, K., Garvey, W.T. & Martins, C., 2024, ‘Strengths and limitations of BMI in the diagnosis of obesity: What is the path forward?’, *Current Obesity Reports* 13(3), 584–595. 10.1007/s13679-024-00580-1 38958869 PMC11306271

[CIT0040] Tan, S.Y., Poh, B.K., Sekartini, R., Rojroongwasinkul, N., Tran, T.N., Wong, J.E. et al., 2024, ‘South East Asian Nutrition Surveys (SEANUTS) II – A multi-country evaluation of nutrition and lifestyle indicators in children aged 12 years and below: Rationale and design’, *Public Health Nutrition* 27(1), e150. 10.1017/S1368980024000910 38639132 PMC11617418

[CIT0041] Tydeman-Edwards, R., Van Rooyen, F.C. & Walsh, C.M., 2018, ‘Obesity, undernutrition and the double burden of malnutrition in the urban and rural southern Free State, South Africa’, *Heliyon* 4(12), e00983. 10.1016/j.heliyon.2018.e00983 30534616 PMC6278724

[CIT0042] University of Cape Town & University of Michigan, 2012, *Cape Area Panel Study 2002–2009, Waves 1–5* (Version 1) [Dataset], DataFirst. 10.25828/yjm7-ap38

[CIT0043] Van Dyk, H. & White, C.J., 2019, ‘Theory and practice of the quintile ranking of schools in South Africa: A financial management perspective’, *South African Journal of Education* 39(Suppl. 1), a1820. 10.15700/saje.v39ns1a1820

[CIT0044] World Health Organization (WHO), 2007, *Growth reference data for 5–19 years*, viewed 19 August 2025, from https://www.who.int/tools/growth-reference-data-for-5to19-years

[CIT0045] World Health Organization (WHO), 2011, *Waist circumference and waist-hip ratio: Report of a WHO expert consultation, Geneva, 8-11 December 2008*, WHO, Geneva, viewed 22 May 2024, from https://www.who.int/publications/i/item/9789241501491

[CIT0046] World Health Organization (WHO), 2021, *Obesity and overweight*, viewed 19 August 2025, from https://www.who.int/news-room/fact-sheets/detail/obesity-and-overweight.

[CIT0047] Wrottesley, S.V., Pedro, T.M., Fall, C.H.D. & Norris, S.A., 2019, ‘A review of adolescent nutrition in South Africa: Transforming adolescent lives through nutrition initiative’, *South African Journal of Clinical Nutrition* 33(4), 94–132. 10.1080/16070658.2019.1607481

